# Postprandial Aminoacidemia Following the Ingestion of Alternative and Sustainable Proteins in Humans: A Narrative Review

**DOI:** 10.3390/nu17020211

**Published:** 2025-01-08

**Authors:** Mohammed Ahmed Yimam, Martina Andreini, Sara Carnevale, Maurizio Muscaritoli

**Affiliations:** 1 Department of Science, Technology and Society, University School for Advanced Studies IUSS Pavia, 27100 Pavia, Italy; 2 Department of Translational and Precision Medicine, Sapienza University of Rome, 00185 Rome, Italy; martina.andreini@uniroma1.it; 3 Department of Public Health, College of Health Science, Woldia University, Woldia P.O. Box 400, Ethiopia; 4 Belcolle Hospital, 01100 Viterbo, Italy; carnevale.sara1@gmail.com

**Keywords:** algae-derived, plant-derived, fungi-derived, insect-derived, aminoacidemia, sarcopenia, aging, chronic diseases, alternative protein sources, sustainability

## Abstract

There is a pressing need to expand the production and consumption of alternative protein sources from plants, fungi, insects, and algae from both nutritional and sustainability perspectives. It is well known that the postprandial rise in plasma amino acid concentrations and subsequent muscle anabolic response is greater after the ingestion of animal-derived protein sources, such as dairy, meat, and eggs, than plant-based proteins. However, emerging evidence shows that a similar muscle anabolic response is observed—despite a lower and slower postprandial aminoacidemia—after the ingestion of alternative protein sources compared with animal-derived protein sources. Therefore, a comprehensive analysis of plasma amino acid kinetics after the ingestion of alternative protein sources would play a significant role in recognizing and identifying the anabolic properties of these protein sources, allowing for the implementation of the best nutritional intervention strategies, contributing to more sustainable food production, and developing new medical nutritional products with optimal impacts on muscle mass, strength, and function, both in terms of health and disease. Therefore, this narrative review is focused on postprandial amino acid kinetics (the area under the curve, peak, and time to reach the peak concentration of amino acids) based on experimental randomized controlled trials performed in young and older adults following the ingestion of different novel, sustainable, and alternative protein sources.

## 1. Introduction

**Figure 1:**
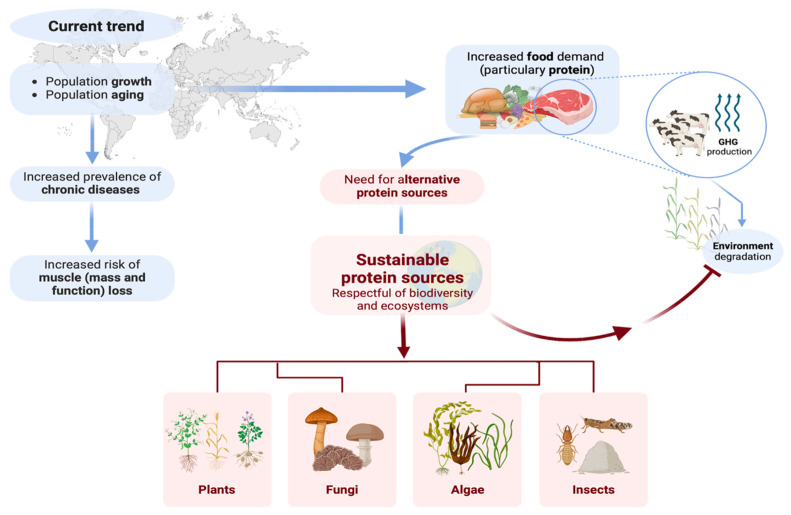
The relationship between the projected population growth and the need for sustainable protein sources (prepared using BioRender.com).

By the year 2050, the global population is projected to reach nearly 10 billion [1]. Consequently, food demand is expected to increase by 56%, and the demand for animal-derived protein is anticipated to double [2,3]. Owing to these demand-side drivers, food systems are changing worldwide, accounting for 19–29% of the total global anthropogenic greenhouse gas emissions [4]. Specifically, the intensive production and consumption of animal-derived proteins significantly contribute to an increase in greenhouse gas emissions, exacerbate the depletion of natural resources (e.g., land and fresh water), amplify terrestrial biodiversity loss [3,5,6,7,8], increase exposure to non-communicable and zoonotic diseases, and drive the escalation of antimicrobial resistance [9,10,11] (Figure 1). As a result, the concept of a sustainable diet—i.e., a diet that respects biodiversity and ecosystems and is culturally acceptable, affordable, and, at the same time, nutritionally adequate, safe, and healthy—has been emphasized by the Food and Agriculture Organization of the United Nations (FAO) from both a nutritional and a sustainability perspective [12]. Promoting sustainable protein diets derived from plant, fungi, edible insects, and algae benefits human health and environmental sustainability [13].

Scaling up the production of sustainable protein sources creates a valuable opportunity to assess their role in human nutrition [14]. The complex landscape of the protein transition envisions a shift from a diet rich in animal proteins to one rich in alternative protein sources [15,16,17]. The global shift toward the production and consumption of alternative protein sources is gaining significant attention [6,18,19]; therefore, considering the protein quality of these protein sources is essential [20]. It has been well established that a diet containing high-quality protein diet prevents protein-energy malnutrition [21], enhances bodybuilding for athletes engaged in training [22], and mitigates sarcopenia (loss of muscle mass, muscle strength, and/or function) [23,24,25]. Therefore, the identification of high-quality protein from novel, sustainable, and alternative protein sources is an indispensable step in this transition.

The nutritional quality of different protein sources in human nutrition is commonly measured using amino acid (AA) scoring. In 1989, FAO/WHO introduced the Protein Digestibility Corrected Amino Acid Score (PDCAAS) to quantify dietary protein quality; in 2011, this was replaced by the Digestible Indispensable Amino Acid Score (DIASS) due to advancements in our understanding of population protein requirements and the ileal digestibility of individual AAs [26,27]. According to the DIAAS system, protein quality is categorized as follows: <75% (no quality claim); 75–99% (high-quality protein); and ≥100% (excellent quality protein) [26]. Therefore, high-quality dietary protein should supply the appropriate pattern of essential amino acids (EAA; leucine, isoleucine, methionine, histidine, phenylalanine, lysine, tryptophan, valine, and threonine), as they cannot be endogenously synthesized by the body, making their intake from dietary protein sources absolutely necessary [28].

The presence of limiting AAs after the ingestion of a protein source can reduce the effectiveness of the post-absorption protein synthesis rate. After the meal, the proteins undergo chemical and mechanical digestion in the mouth, stomach, and duodenum until they are absorbed into the intestinal lumen as individual AAs. Splanchnic tissues, mainly the intestine and liver, play an important role in the absorption of dietary AAs and their subsequent release into the peripheral tissues, such as muscles [29]. 

**Figure 2:**
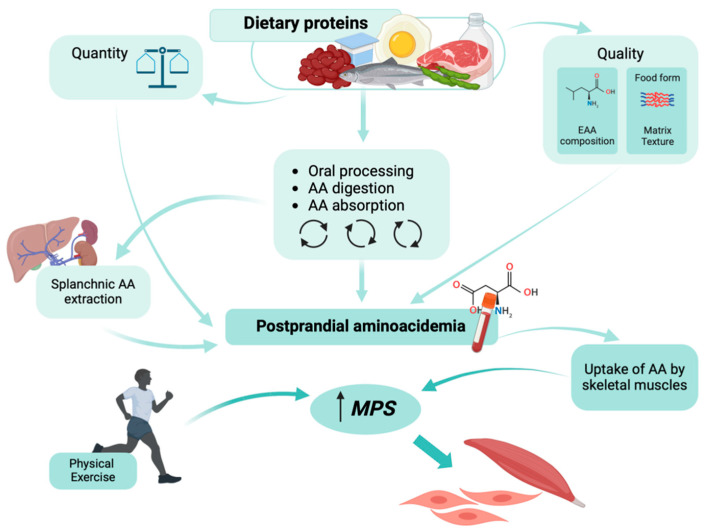
Factors regulating postprandial aminoacidemia and muscle protein synthesis (MPS) following a protein meal in normal conditions (prepared using BioRender.com).

In addition, the food form (beverage versus solid, particularly in older adults), food matrix, amount, and type of protein, as well as the rate of protein digestion, influence the postprandial AA absorption kinetics (amount, rate of absorption, and overall availability of AAs in the circulation), ultimately playing a crucial role in determining the muscle anabolic response [29,30,31,32,33,34] (Figure 2). Protein sources that produce large and rapid increases in peripheral EAA concentrations (e.g., whey protein) have been shown to be effective in improving muscle and whole-body protein synthesis [35].

Therefore, a comprehensive look into the postprandial plasma AA kinetics following the ingestion of alternative protein sources plays a central role in identifying the anabolic properties of the protein sources, allowing for the implementation of optimal nutrition intervention strategies, contributing to more sustainable food production, and developing nutrition products designed to maximize their impact on muscle anabolism. The present narrative review focuses on the key aspects of postprandial amino acid kinetics, such as the peak AA concentration, the time to reach the peak AA concentration, and overall postprandial AA availability, which is expressed as the area under the curve (AUC), following the ingestion of alternative protein sources in vivo in adult populations based on randomized controlled trials (RCTs).

## 2. Methods

PubMed database and Google Scholar were used from their inception up to 17 November 2024 to search published articles related to postprandial aminoacidemia after the ingestion of alternative sources of protein, using the following key search terms: plant protein OR fungi protein OR mycoprotein OR algae protein OR spirulina OR chlorella OR insect protein OR protein blend OR postprandial aminoacidemia OR plasma amino acid concentrations OR aminoacidemia OR muscle protein synthesis AND randomized controlled trials (parallel or crossover trial). Both forward and backward citation searches on the selected references were applied to ensure the inclusion of all relevant studies. The present review includes articles published in English, adults aged ≥18 years , and orally ingested protein sources (excluding intravenous feeding).

## 3. Protein Ingestion and Muscle Anabolism

Muscles are the main reservoir of protein within the body and undergo high rates of protein turnover [36]. Therefore, food intake, particularly protein ingestion, is an important regulator of muscle protein metabolism. It is extremely relevant to reverse the negative protein balance (when muscle protein breakdown exceeds MPS) that predominates during post-absorptive periods through protein ingestion and exercise [36]. Mostly, feeding-induced increases in the plasma concentrations of AAs stimulate MPS [36,37]. In healthy young adults, protein meal ingestion leads to an increase in the plasma concentrations of AA and hormones, resulting in a spike in protein synthesis and a reduction in muscle protein degradation rates. In simple terms, the skeletal muscle tissue in young adults is highly responsive to protein intake [38]. The recommended dietary allowance (RDA) for healthy adults to meet the metabolic needs under normal conditions is 0.8 g/body weight (BW)/day, but this amount varies depending on the age, pregnancy and disease conditions [39].

To maximize MPS, older adults and/or people with chronic diseases require a higher protein intake than younger individuals. Explicitly, younger individuals can maximize MPS with approximately 0.25 g protein·kg BW^−1^ per meal, whereas older individuals require approximately 0.40 g protein·kg BW^−1^ per meal [40] due to ‘anabolic resistance’, which is defined as the inability of the muscles to mount a robust increase in MPS in response to AA and exercise [41,42,43,44,45,46,47,48]. The mechanisms explaining the ‘anabolic resistance’ of elderly muscle to protein provision are not clear due to inconsistent findings across studies [41,43,49]; however, impairment in digestion and/or absorption after a protein meal may partly contribute to the blunted anabolic response.

Consequently, to maintain and regain muscle mass and function in healthy older adults, the PROT-AGE study group recommends an average dietary protein intake at least in the range of 1.0–1.2 g/kg/day. In addition, the per-meal anabolic threshold of dietary protein/amino acid intake in older individuals needs to be at least 25 to 30 g of protein, containing 2.5 to 2.8 g of leucine [50]. In contrast, intake of a 20 g high-quality protein meal in young adults maximally stimulates MPS for several hours, particularly during post-exercise recovery [51,52].

Furthermore, the Society for Sarcopenia, Cachexia and Wasting Diseases (SCWD) recommends a protein intake of 1.0–1.5 g/kg/day in combination with adequate exercise to prevent muscle loss in older people [44]. Therefore, an adequate distribution of high-quality protein quota at meals (0.4 g/kg/meal) in conjunction with physical exercise is an effective strategy to counteract sarcopenia [40]. In short, regardless of age, protein ingestion should undergo digestion and AA absorption to participate in metabolism.

## 4. Postprandial Aminoacidemia Following the Ingestion of Sustainable Protein Sources

### 4.1. Postprandial Aminoacidemia Following the Ingestion of Plant-Derived Proteins

Plant-derived proteins are environmentally sustainable and optimal for health [3,16,53,54,55,56,57]; however, they are anabolically inferior to animal-derived proteins [30,58,59,60,61,62,63,64] due to their lower digestibility and bioavailability, as well as their relative deficiency of one or more EAA [65,66,67,68]. In fact, the AA composition differs across plant protein sources [64]. In particular, cereals (e.g., wheat and corn) are deficient in lysine, while legumes (e.g., peas, broad beans, and lentils) are lacking methionine and cysteine. Interestingly, potato and soybeans contain all the AAs necessary for human nutrition according to the joint report of WHO/FAO/UNU expert consultations for protein requirements [69]. Therefore, a judicious selection of plant-derived protein sources is necessary to obtain an optimal EAA profile.

Furthermore, to solve the EAA deficiencies of plant-derived protein sources, different strategies have been identified, such as fortifying specific AAs (leucine, lysine, and/or methionine), breeding selective plants, and blending different plant proteins [70,71,72,73,74,75,76,77,78,79]. Increasing the amount of plant-based whole foods (e.g., consuming 90 g of soy or 1050 g of potatoes to obtain 20 g of high-quality protein) could also be a measure to increase the AA profile and overcome the lower anabolic capacity of plant-derived proteins; however, this strategy may not be feasible and realistic [71,80]. As a result, the food industry has been producing different plant protein concentrates, isolates, and hydrolysates [81,82] to improve protein quality [83,84].

The ingestion of plant-derived proteins, such as soy protein isolate [30,85,86,87,88], potato protein concentrate [89,90], pea protein concentrate [91], and wheat–corn–pea blend protein [92], showed marked differences in the postprandial AA kinetic parameters. A study measuring the postprandial aminoacidemia for 180 min and simultaneously determining the MPS response after the ingestion of soy protein isolate (22.2 g), casein (21.9 g), and whey protein hydrolysate (21.4 g) in young healthy adults revealed that soy protein isolate showed a lower peak and slower rise in leucine, BCAA, and EAA concentrations compared to whey protein hydrolysate (Table 1). However, soy protein showed a higher amplitude and a faster rise in leucine, BCAA and EAA concentrations than casein [30]. Notably, the proteins compared in this study have equal amounts of EAA (10 g). However, the leucine amount is 2.3 g in whey protein hydrolysate and 1.8 g in soy protein isolate and casein [30].

Given that equal amounts of EAA and approximately equivalent grams of protein were provided in this study, the observed differences in the AA pattern in circulation are quite surprising, which may be partly attributed to marked variations in the rate of digestion among protein sources. It is well known that both soy and whey protein are rapidly digested proteins compared to casein; thus, a greater and rapid aminoacidemia was observed in both proteins relative to casein. In the end, differences in leucine and EAA kinetics in the circulations resulted in MPS differences among the protein sources (whey > soy > casein), particularly after exercise [30]. However, why did the soy and whey proteins show marked differences in the amino acid kinetics and MPS responses, despite both proteins being rapidly digested? Although the answer was not provided by the above study [30], previous studies have revealed that AAs from soy are mainly extracted in the splanchnic tissues and converted to urea to a greater extent than AAs from dairy-based proteins [93,94].

In support, postprandial aminoacidemia measured in the elderly for 4 h after the ingestion of graded doses of soy protein isolate (20 g or 40 g) showed a lower amplitude and a slower rise in leucinemia compared to graded doses of whey protein isolate (20 g or 40 g) [85]. Moreover, higher AA oxidation was observed after the ingestion of 20 g of soy protein versus 20 g of whey protein. The lower postprandial leucinemia and greater rates of amino acid oxidation following the ingestion of soy versus whey protein resulted in the inability to stimulate MPS at rest [85]. When coupled with exercise, only 40 g of soy protein isolate modestly stimulates MPS [85]. This clearly highlights that the protein dose and protein source influences the postprandial AA kinetics (dose–response relationship). Furthermore, plant protein formulations designed to stimulate MPS in the elderly populations need to consider the optimal protein doses and adequate amount of leucine. 

Recent studies have shown that the postprandial aminoacidemia measured for 5 h after a single bolus ingestion of either 30 g of potato protein concentrate or 30 g of pea protein concentrate versus an equal amount of milk protein comparators in young healthy adults showed marked differences in the AA kinetics [89,91]. Smaller and delayed increases in leucine, lysine, methionine, and EAA concentrations were observed after the ingestion of potato and pea concentrate compared to the milk protein [89,91]. The observed low postprandial availability of AA after ingesting pea and potato protein concentrates may partly be due to the attenuated rate of digestion and the retention of AA in the splanchnic tissues. Moreover, differences in the AA composition of the pea protein concentrate versus milk protein may have contributed to the lower postprandial appearance of AA in the circulation [91]. However, the observed lower aminoacidemia after the ingestion of potato and pea protein concentrates compared with milk protein did not result in MPS rate differences among the protein sources at rest or following exercise [89,91]. The reason for this is not entirely clear; however, the overall postprandial plasma availability of leucine and EAA after the ingestion of 30 g of plant protein concentrates may be sufficient to trigger MPS machinery, similar to the effect of 30 g of milk protein.

Furthermore, postprandial aminoacidemia measured in healthy young adult males over 5 h after ingesting 30 g of wheat–corn–pea blend protein showed lower and slower increases in lysine, methionine, and EAA concentrations compared to 30 g of milk protein [92]. The lower availability of these AAs after ingestion of the protein blend may be due to the lower EAA (7.4 g versus 9.8 g), lysine (0.7 g versus 2 g), and methionine (0.4 g versus 0.7 g) contents compared to those in the milk protein. Surprisingly, a lower and slower leucine concentration was observed after ingesting the protein blend compared to milk protein despite an equal amount (2.4 g versus 2.4 g) of leucine being found in both protein beverages. In the end, the attenuated postprandial aminoacidemia did not result in differences in MPS between the protein sources [92].

The plasma AA concentrations measured 3 h after consuming 30 g of a novel aquatic plant called Mankai (a newly developed high-protein strain of *Wolffia globosa* duckweed) versus 30 g of soft cheese showed similar EAA concentrations [95]. From ecological and nutritional standpoints, Mankai may serve as an alternative to animal protein; however, further anabolic studies are required.

In summary, based on the available evidence [30,85,89,91,92], the following concepts may be important for the food industry and nutrition companies in producing more economically and environmentally sustainable plant-based protein products: (1) the AA composition and the rate of protein digestion mainly influence the postprandial AA kinetics; (2) ingesting an equal amount of plant protein isolates, concentrates, or plant-derived protein blends compared with animal protein sources shows a similar muscle anabolic response despite differences in the postprandial AA kinetics; (3) plant-only protein blends are capable of stimulating MPS to the same extent as animal-derived proteins ; (4) pea, soy, and potato protein might be viable alternatives to animal protein, and could have an enormous application in sports nutrition; (5) further studies on the postprandial aminoacidemia and/or muscle anabolic response after the ingestion of plant-only blend proteins, concentrates, and isolates is warranted to scale up the use of plant protein sources.

**Table 1:** Randomized controlled trial studies reported postprandial aminoacidemia following ingestion of alternative and sustainable protein sources in adults.

Alternative Protein Source	Name of Protein	Study Participant	Duration	Intervention Products in Protocol	Postprandial Aminoacidemia
Plant	Soy protein [30]	18 men; 22.8 ± 3.9 years	3 h	21.4 g of whey protein hydrolysate , 22.2 g of soy protein isolate, and 21.9 g of casein	- All proteins stimulated increases in EAA (whey ≈ soy > casein) and leucine (whey > soy >casein) by 30 min - At 60 min, EAA and leucine were increased in whey > soy > casein. - AUC of blood leucine was ↑ in whey (whey > soy > casein) - MPS rate was ↑ in whey and soy compared to casein at rest (whey ≈ soy > casein) and after resistance exercise (whey > soy > casein).
	Soy protein [85]	30 men; 71 ± 5 years	4 h	20 g or 40 g of soy protein isolate, and 20 g or 40 g of whey protein isolate	- 20 g and 40 g of soy protein isolate showed a ↓ peak in leucinemia. - T_max_ of leucinemia occurred at 1–1.5 h for 20 g of soy and 20 g and 40 g of whey protein, but ~1.5–2 h for 40 g of soy protein isolate - AUCs of leucine, BCAA, EAA, and TAA were ↑ in 40 g versus 20 g of whey - No significant differences in AUCs of leucine, BCAA, EAA between 40 g and 20 g of soy protein isolate - Ingestion of 20 g and 40 g of soy protein isolate does not stimulate increased rates of MPS under resting conditions in the elderly.
	Potato protein [89]	24 Men; 24 ± 4 years	5 h	30 g of potato protein concentrate and 30 g of milk protein concentrate	- EAA concentrations and its peak values were ↓ in 30 g of potato versus 30 g of milk protein - T_max_ of plasma leucine, lysine, and methionine were longer for potato protein than milk protein - Peak TAA concentrations were 37% ↓ in potato protein versus milk - T_max_ for TAA concentrations were 118 and 43 min after potato and milk protein ingestion, respectively - MPS rate was similar between interventions
	Plant-derived protein blend(wheat, corn, and pea) [92]	24 males; 24 ± 4 years	5 h	30 g of plant-derived protein blend, and 30 g milk protein	- Faster and greater increases in leucine, lysine, methionine, and EAA after milk than plant-derived protein blend ingestion - Milk protein had ↑ AUC of leucine and EAA for 300 min. - Glycine plasma availability was ↑ in plant-derived protein blends for 5 h - MPS rate was similar between protein sources
Fungi	Mycoprotein [96]	20 men; 22 ± 1 years	4 h	70 g of mycoprotein, and 31 g of milk protein. Both proteins contains equal amounts of leucine (2.5 g)	- BCAA peaked more rapidly following milk protein (T_max_: 82.5 ± 15 min) than mycoprotein (T_max_: 103 ± 10 min) - Plasma leucine, phenylalanine and tyrosine were significantly ↑ in milk than mycoprotein. - AUCs of leucine concentrations were 19 ± 8% ↑ in milk than mycoprotein - Surprisingly, MPS rate was greater in Mycoprotein
	Mycoprotein [97]	24 male; 21 ± 2 years	4 h	70 g of mycoprotein whole food matrix (MYC), and 38.2 g of a protein concentrate obtained from mycoprotein. Both proteins contain equal amounts of leucine (2.5 g)	- Plasma BCAA, TAA, and EAA displayed ↑, rapid and sustained after ingestion of PC compared to MYC - Leucine and isoleucine remained elevated longer (between 150 and 240 min) in the MYC than PC - MPS rate was similar between interventions
	Blend of mycoprotein and pea protein [98]	33 men and women; 21 ± 1 years	4 h	25g of mycoprotein (MYC), 25 g of pea protein (PEA), 25 g of mycoprotein and pea protein blend (BLEND)	- Plasma BCAA, EAA, NEAA, and TAA displayed rapid increase following ingestion of PEA and BLEND compared to MYC. - Plasma leucine concentrations peaked more rapidly in the PEA and BLEND conditions than MYC - Plasma concentration and AUC of methionine was ↓ following ingestion of PEA and ↑ following ingestion of MYC and BLEND (MYC > BLEND > PEA). - MPS rate was similar (MYC = BLEND = PEA)
Insects	Lesser mealworm-derived protein [99]	24 men; 23 ± 3 years	5 h	30 g of lesser mealworm protein and 30 g of milk protein	- Plasma C_max_ of leucine and phenylalanine were ↑ for milk than mealworm at all time points, except *t* = 0 and 120 min - Peak concentrations of leucine, and phenylalanine ↑ for milk than mealworm - Plasma tyrosine concentrations were ↑ for mealworm than for milk from *t* = 60 through 300 min - MPS rates were similar between protein sources
	Lesser mealworm protein isolate [100]	6 men; 18–30 years	2 h	30.5 g of lesser mealworm protein isolate, 27.8 g of whey protein isolate, and 28.7 g ofsoy protein isolate	- Ingestion of whey, soy, and lesser mealworm protein isolate increased blood concentrations of EAA, BCAA, and leucine over a 120 min period (whey > insect = soy). - Lesser mealworm protein induced blood AA concentrations similar to soy protein. - MPS rate was not determined in the study (open for future research)
	Cricket protein powder [101]	50 samples; 18–30 years	4 h	Cricket protein powder (0.25 g/kg fat-free mass), whey protein concentrate, and pea protein isolate (0.25 g/kg fat-free mass)	- BCAA, and leucine concentrations were significantly higher at 20, 40, and 60 min after the ingestion of whey than pea, and cricket. - AUC of BCAA was different between whey and pea protein but not cricket protein. - AUCs of TAA did not differ among groups (whey = pea = cricket). - AUCs of EAA and leucine were significantly different among protein groups (whey > pea > cricket) - No significant differences were observed in mTORC1 signalling among the protein sources
	Cricket-derived protein [102]	24 males; 23 ± 4 years	5 h	25 g of cricket-derived protein and 25 g of beef-derived protein	- Increased AUCs of leucine, BCAA, and EAA for cricket - AUCs of NEAA and TAA of cricket was ↓ compared to beef. The AA plasma level peaked earlier (at 60–80 min) for beef-derived protein compared to cricket (at 90–100 min)
Algae	Spirulina and chlorella [103]	36 male: 22 ± 3 years	4 h	25 g of spirulina, 25 g of chlorella, and 25 g of mycoprotein (MYC)	- The rate and peak concentrations of TAA and EAA were significantly ↑ in spirulina compared to MYC and chlorella (spirulina > mycoprotein > chlorella). - Stimulation of MPS rate was similar.

↓: lower; ↑: higher; ≈: approximately equal; AUC: area under the curve; BCAA: branched chain amino acids; EAA: essential amino acids; NEAA: non-essential amino acids; TAA: total amino acids; C_max_: Maximum concentration; T_max_: time to reach peak concentration.

### 4.2. Postprandial Aminoacidemia Following the Ingestion of *Fungi-Derived Protein*

Fungi are an emerging protein source due to their high-quality protein components, high production efficiency, and their role as champions in promoting a circular economy [104]. Mycoprotein is a popular fungal protein that has been applied in sports nutrition for stimulating MPS and muscle reconditioning after exercise [96,97,98,105]. At the basic level, mycoprotein is a whole food produced from the continuous fermentation of filamentous fungus, *Fusarium venenatum* [106]. Mycoprotein contains high-quality proteins, carbohydrates, fats, fiber, vitamins, and minerals [104]. The protein content in mycoprotein is highly rich in EAA (41% of the total protein) and it has a PDCAAS of 0.996 [106,107].

A study measuring postprandial aminoacidemia for 4 h after the ingestion of graded doses of mycoprotein (40 g, 60 g, and 80 g) showed lower and slower EAA patterns compared to 20 g of milk protein ingestion [108]. In support, ingesting mycoprotein (70 g) compared to milk protein (31 g) showed lower and slower but sustained increases in leucine and EAA concentrations even though both protein supplements are matched in leucine content [96] (Table 1). The carbohydrates found in mycoprotein could contribute to reduced leucinemia. Studies have shown that carbohydrate co-ingestion with protein reduces the magnitude and amplitude of leucine concentrations without attenuating the anabolic response of the protein [109,110,111].

A handful of studies [97,98,105] have shown that ingesting BCAA-enriched mycoprotein, protein concentrate derived from mycoprotein, and a blend of mycoprotein with pea protein increases postprandial plasma EAA concentrations. Specifically, ingesting 35 g of BCAA-fortified mycoprotein and 38.2 g of protein concentrate of mycoprotein resulted in a greater amplitude and rapid concentrations of EAA compared with 70 g of mycoprotein ingestion [97,105]. In the same vein, ingesting 25 g of a protein blend of mycoprotein with pea resulted in greater, faster increases in leucine and EAA concentrations compared with ingesting 25 g of mycoprotein or 25 g of pea protein [98].

In summary, a large dose (70 g) of mycoprotein ingestion stimulates MPS greater than 31 g of milk protein in young, healthy adults despite having lower postprandial EAA concentrations [96]. This finding may not be easily transferable to the clinical setting in which consuming large and highly satiating mycoprotein [112,113] is challenging, particularly for anorexic older adults and sarcopenic and cachexic patients. Therefore, further research on the postprandial AA kinetics and anabolic effects of protein concentrate from mycoprotein and BCAA-enriched mycoprotein in the elderly is warranted.

### 4.3. Postprandial Aminoacidemia Following the Ingestion of *Insect-Derived Protein*

**Figure 3:**
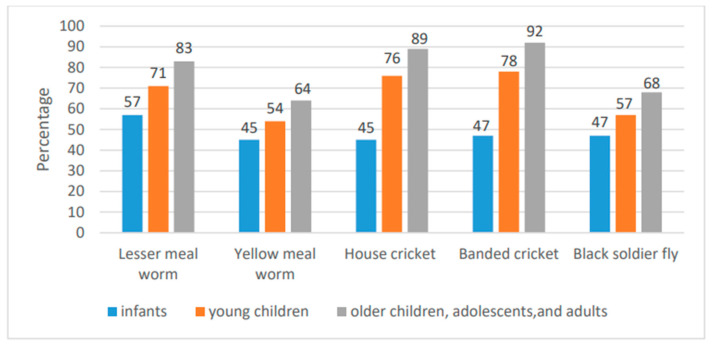
Calculated DIAAS of edible insects across the recommended FAO AA scoring pattern.

Edible insects could be a sustainable, alternative dietary protein source because they have a higher protein density, result in lower greenhouse gas emissions, and require less water and land compared to cattle [114,115,116,117]. High-quality proteins can be obtained from edible insects, as measured by the DIASS [118] (Figure 3). For instance, house and banded crickets are categorized as a good-quality protein source for all age-related scoring patterns (infants, children, adolescents, and adults), whereas lesser mealworm (*Alphitobius diaperinus*) is a good-quality protein source for older children, adolescents, and adults [118]. In addition, protein derived from lesser mealworm has an amino acid composition similar to that of milk and meat [115,116].

A study conducted on young male adults participating in resistance training showed lower and slower increases in EAA, leucine, and phenylalanine concentrations, observed for a 5 h period after ingesting 30 g of lesser mealworm-derived protein isolate compared to 30 g of milk protein. However, the MPS rate was not statistically different between the protein sources at rest and during recovery from resistance-type exercise [99]. The lower aminoacidemia following mealworm ingestion may be explained by the lower digestibility of mealworm compared with milk protein. However, the lower and slower increases in the EAA, leucine, and phenylalanine concentrations did not impair the subsequent MPS response after lesser mealworm ingestion. The rationale is not completely clear but may be explained by the fact that the available EAA leucine and phenylalanine concentrations may have reached the required amount to initiate the MPS. Further studies in young and older adults are needed to clearly elucidate this hypothesis.

Moreover, ingesting 30.5 g of lesser mealworm protein isolate increased the leucine, BCAA, and EAA concentrations similarly to ingesting 27.8 g of soy protein isolate during a 120 min postprandial period [100]. However, it would be important to assess this effect during a 3–5 h postprandial period to clearly understand the full picture of the digestibility and bioavailability of the protein sources. Recently, a study evaluated the effect of ingesting 25 g of cricket-derived protein compared to 25 g of beef-derived protein on aminoacidemia for 5 h, showing that the leucine, BCAA, and EAA concentrations were greater after cricket protein ingestion than beef-derived protein ingestion [102]. However, the observed leucine, BCAA, and EAA concentrations appeared more rapidly for the beef-derived protein [102] (Table 1). Another study showed that higher and more rapid concentrations of EAA, including leucine, occurred after the ingestion of whey compared to cricket and pea protein; however, no differences in the mammalian target of rapamycin complex 1 (mTORC1) signaling were observed among the protein sources [101]. The differences observed in leucine might be due to the different protein sources’ leucine contents, which were 0.98 g, 1.19 g, and 1.62 g for cricket, pea, and whey protein, respectively [101]. The similarity in the mTORC1 signaling pathway in this study may be partly explained by the bioavailability of leucine and EAA from all protein sources having reached a sufficient level to similarly initiate the signaling pathway. However, the data obtained in this study warrant further investigation. 

In summary, acute studies on insect-derived proteins have shown markedly different AA kinetics and muscle anabolic responses in young adults [99,101]. These findings lead to further inquiry in older adults on the wider applications of insect-based proteins as a sustainable, alternative protein source for human nutrition.

### 4.4. Postprandial Aminoacidemia Following the Ingestion of Algae-Derived Protein

Algae are a sustainable protein source that provide an optimal nutrients and have a small environmental footprint [119,120,121]. Chlorella (*Chlorella vulgaris*) and spirulina (*Arthrospira platensis*) are commercially available algae-derived proteins for human consumption that have in vitro protein digestibility (their in vivo ileal digestibility is unknown) of 80% and 75%, respectively [120,122].

Although evidence on postprandial aminoacidemia following the ingestion of algae-derived protein is limited, a recent study examined the effect of spirulina and chlorella versus mycoprotein ingestion on postprandial AA kinetics and MPS rates in young adults [103]. After ingesting equal amounts (25 g) of spirulina, chlorella, and mycoprotein, the plasma leucine, EAA, and TAA peak concentrations were significantly higher after spirulina supplementation than the others (spirulina > mycoprotein > chlorella) [103]. Despite higher postprandial AAs being available in spirulina (spirulina > mycoprotein > chlorella), the MPS rate was similar among the ingested protein supplements [103]. This evidence further supports the concept that differences in the blood concentrations of EAA and leucine might not always lead to differences in MPS [123,124,125,126]. More research is needed on postprandial AA aminoacidemia and anabolic response following the ingestion of algae-derived proteins at different ages, in both young and old adults.

## 5. Conclusions

Based on the current narrative review, we conclude that the ingestion of proteins derived from plants, fungi, insects, and algae elicits strikingly different postprandial plasma AA responses, which suggests further work is needed to assess the ability of these protein sources to induce an appropriate muscle anabolic response in humans and, in particular, to prevent or treat age-related or disease-related sarcopenia. Protein blends might more efficiently enhance postprandial AA concentrations, ultimately stimulating MPS. Research on protein sources alternative to meat and dairy will not only contribute to more sustainable food production to meet future global dietary protein demands but will also help develop new medical nutrition products that have an optimal impact on muscle mass and function in both health and disease.

Future work will need to focus on investigating postprandial AA kinetics and muscle anabolic responses after ingesting protein blends, specific AA fortified proteins, whole foods, and mixed meals derived from alternative protein sources in different age groups, genders, populations, and health conditions.
